# MOTS-c is an effective target for treating cancer-induced bone pain through the induction of AMPK-mediated mitochondrial biogenesis: The role of MOTS-c in bone cancer pain

**DOI:** 10.3724/abbs.2024048

**Published:** 2024-05-08

**Authors:** Long Yang, Miaomiao Li, Yucheng Liu, Yang Bai, Tianyu Yin, Yangyang Chen, Jinhong Jiang, Su Liu

**Affiliations:** 1 Jiangsu Province Key Laboratory of Anesthesiology Jiangsu Province Key Laboratory of Anesthesia and Analgesia Application Technology NMPA Key Laboratory for Research and Evaluation of Narcotic and Psychotropic Drugs Xuzhou Medical University Xuzhou 221004 China; 2 Department of Anesthesiology the Affiliated Hospital of Xuzhou Medical University Xuzhou 221018 China

**Keywords:** MOTS-c, AMPK, bone cancer pain, mitochondrial, osteoclast

## Abstract

Bone cancer pain (BCP), due to cancer bone metastasis and bone destruction, is a common symptom of tumors, including breast, prostate, and lung tumors. Patients often experience severe pain without effective treatment. Here, using a mouse model of bone cancer, we report that MOTS-c, a novel mitochondrial-derived peptide, confers remarkable protection against cancer pain and bone destruction. Briefly, we find that the plasma level of endogenous MOTS-c is significantly lower in the BCP group than in the sham group. Accordingly, intraperitoneal administration of MOTS-c robustly attenuates bone cancer-induced pain. These effects are blocked by compound C, an AMPK inhibitor. Furthermore, MOTS-c treatment significantly enhances AMPKα _1/2_ phosphorylation. Interestingly, mechanical studies indicate that at the spinal cord level, MOTS-c relieves pain by restoring mitochondrial biogenesis, suppressing microglial activation, and decreasing the production of inflammatory factors, which directly contribute to neuronal modulation. However, in the periphery, MOTS-c protects against local bone destruction by modulating osteoclast and immune cell function in the tumor microenvironment, providing long-term relief from cancer pain. Additionally, we find that chronic administration of MOTS-c has little effect on liver, renal, lipid or cardiac function in mice. In conclusion, MOTS-c improves BCP through peripheral and central synergistic effects on nociceptors, immune cells, and osteoclasts, providing a pharmacological and biological rationale for the development of mitochondrial peptide-based therapeutic agents for cancer-induced pain.

## Introduction

Bone cancer pain (BCP) is a common clinical symptom of cancer patients, who often experience severe pain without effective treatment [ 1, 2] . Tumors, such as prostate, breast, and lung cancers, have a strong tendency for bone metastasis and bone destruction [ 1, 3] . Mounting evidence has indicated that bone metastasized tumor cells generate pain by releasing inflammatory factors, growth factors, and algogenic substances, which can induce sensitization and activation of the nerve fibers that innervate bone [ 4– 6] . Furthermore, tumor cells can also directly activate sensory nerve fibers, which in turn contribute to peripheral and central sensitization [ 2, 7] . Invasion of bone by tumor cells evokes infiltration of immune cells, including macrophages, T cells and granulocytes, and produces and releases pro-inflammatory mediators ( *e*. *g*., IL-1β, IL-6, TNF-α, and CCL5), which induce pain by binding to their receptors in sensory neurons [ 2, 8, 9] . Although BCP is severe and widespread in patients with advanced tumors, the efficacy of currently available drugs for BCP treatment is limited due to numerous unwanted side effects [ 1, 6] . Therefore, it is necessary to find new therapeutic targets for BCP to improve the quality of life of cancer patients.

MOTS-c, or the mitochondrial open reading frame of 12S rRNA type-c, is a recently discovered mitochondrial-derived peptide [10]. Several recent studies have shown that MOTS-c is involved in the regulation of diabetes [ 11, 12] , insulin resistance [ 10, 13] , inflammation [ 14, 15] , aging [16] and osteoporosis [ 17, 18] through the AMP-activated protein kinase (AMPK) pathway. In particular, AMPK has attracted a great deal of attention as a therapeutic target for the regulation of chronic pain [ 19– 21] . The role of metformin in chronic pain has also been reported to be associated with AMPK activity [22]. Our previous study revealed that a single acute treatment with MOTS-c relieves nerve injury-induced neuropathic pain and inflammatory pain [ 23, 24] . However, due to BCP, the severe pain faced by cancer patients in the late stages of the disease, it is difficult to achieve the desired efficacy of most medications [25], such as pregabalin and celecoxib, for treating neuropathic pain and inflammatory pain.

Our previous report showed that treatment with MOTS-c alone inhibits microglial activation in acute spared nerve injury-induced neuropathic pain [26]. BCP is a chronic neuralgia requiring long-term medication, and whether MOTS-c can exert an analgesic effect on BCP is unknown. In the present study, we evaluated the potential effects of MOTS-c on chronic BCP and investigated the underlying regulatory mechanisms involved. We found that mechanical withdrawal markedly decreased in latency 4 days after the injection of Lewis lung cancer (LLC) cells into the medullary cavity. Intraperitoneal (i.p.) injection of MOTS-c produced pronounced and dose-dependent antinociceptive effects in BCP model mice, indicating that MOTS-c may serve as a target for the treatment of bone cancer pain. These findings indicated that MOTS-c may be involved in the regulation of BCP, and understanding the associated regulatory mechanism may provide novel and effective therapeutic targets for BCP treatment.

## Materials and Methods

### Experimental mice

Male C57BL/6 mice (aged 6‒10 weeks, weighing 20‒25 g) were obtained from the Experimental Animal Center of Xuzhou Medical University and were raised in cages in an air filtration system. The animals were maintained under a 12/12-h light/dark cycle (8 am ‒8 pm), and the temperature range was 22–25°C, with free access to food and water. The treatment of the animals in this study was in accordance with the relevant guidelines of the International Federation for the Study of Pain, and all animal care and experimental protocols were approved by the Animal Care and Use Committee of Xuzhou Medical University (permission number: 202208S044). The mice were randomly divided into 5 groups: the sham group (sham), BCP+vehicle group (BCP), BCP+MOTS-c group (BCP+MOTS-c), BCP+morphine group (BCP+morphine), and BCP+Celebrex group (BCP+celebrex). Mice were anesthetized with pentobarbital (50 mg/kg, intraperitoneal) to prepare the spinal cord tissue and dorsal root ganglion (DRG) for western blot analysis and immunobiochemistry. MOTS-c (0.5, 1 and 2 mg/kg), morphine (1, 5 and 10 mg/kg), and celebrex (30 mg/kg) were administered intraperitoneally. The doses used were chosen based on our previous report [23].

### Drugs

MOTS-c was synthesized by a standard Fmoc-based solid phase synthesis methodology as described in our previous report [26]. All amino acids and coupling reagents were purchased from GL Biochem Ltd. (Shanghai, China). Compound C was purchased from MedChemExpress (Shanghai, China), dissolved in 10% DMSO, kept at ‒20°C, and diluted in corn oil immediately before i.p. injection (10 mg/kg). Lipopolysaccharide (LPS) was purchased from Sigma (St Louis, USA) and dissolved in saline (1 μg/μL). Morphine hydrochloride was obtained from Shenyang First Pharmaceutical Factory (Shenyang, China). Celebrex was obtained from Medchemexpress (Cat No. 169590-42-5).

### Animal model of bone cancer pain

A mouse model of BCP was established as previously reported [9]. In brief, LLC cells (Cat No. TCM-C742; Haixing Biosciences **,** Zhaoqing, China) were detach using 0.125% trypsin, collected by centrifugation, and resuspended at a concentration of 2×10 ^5^ cells/μL in sterile phosphate-buffered saline (PBS). Mice were anesthetized with 2% sodium pentobarbital, the superficial skin of the left leg was shaved and sterilized with 75% ethanol, an incision of approximately 1 cm long was made in the distal femur area, and 10 μL of LLC cell suspension was slowly injected into the medullary cavity of the left distal femur using a 20-μL microinjection syringe. The syringe needle was left for 90 s after injection completion to allow the tumor cells to fill the cavity. The injection site was then sealed with sterile bone wax to prevent leakage of tumor cells. The wound was disinfected with antibiotics and then sutured. The sham group was injected with 10 μL of sterile PBS into the left femur.

### Mechanical allodynia and thermal hyperalgesia testing

The paw withdrawal threshold (PWT) was measured using von Frey monofilaments (Stoelting Co., Wood Dale, USA) with the ascending method [27] to assess mechanical allodynia. First, the mice were placed in a plexiglass chamber on a metal mesh floor and allowed at least 30 min to habituate for three consecutive days before the experiment. With sustained pressure to bend the filament for 3 s or elicit a paw withdrawal reflex within 3 s, a series of filaments (2.36, 2.44, 2.83, 3.22, 3.61, 3.84, 4.08, 4.17 and 4.31) were applied to the plantar surface of the hind paw. A total of six responses were recorded, and the 50% withdrawal threshold from the paw was statistically analyzed based on previous methods [ 28, 29] .

Paw withdrawal latency (PWL) was measured using a plantar analgesia meter (Boerni, Tianjin, China) to assess thermal hyperalgesia. In brief, the mice were placed in a plexiglass chamber on a glass plate and allowed at least 30‒60 min to habituate for three consecutive days. The PWL was recorded by an automatic timer and was defined as the duration from the onset of focused radiant heat to the withdrawal of the hind paw. A 20-s cut-off time of heating was used to avoid potential tissue damage. For each mouse, with a 10-min interval, the heat stimulus was repeated three times to determine latency [23].

### Immunofluorescence (IF) assay

Under deep anesthesia, the mice were transcardially perfused with 20 mL of PBS followed by 20 mL of 4% paraformaldehyde (PFA). The whole lumbar spinal cord (SC) at the L4‒L5 segments was then dissected, postfixed overnight at 4°C and cryoprotected in 30% sucrose at 4°C. The embedded blocks were sectioned (20 μm thick). The sections were placed in a small, heat-resistant basket, immersed in retrieval solution (10 mM sodium citrate buffer, pH 6.0) for 5 min, and then stored in PBS at 4°C. At the same time, the femora were removed and then fixed for 48 h in the same fixative at 4°C. After demineralization in EDTA (10%) for 10 days, femur samples were dehydrated in an ascending gradient of ethanol (30%‒100%) followed by paraffin embedding. Serial sections for trabecular bone were obtained from the distal femur at a thickness of 8 μm. Both sections were blocked in a solution containing 1% BSA, 5% donkey serum (Abcam, Cambridge, UK) and 0.3% Triton X-100 at room temperature for 2 h, after which the spinal cord sections were incubated with the following primary antibodies overnight at 4°C: anti-NeuN (1:500; ab177487; Abcam), anti-GFAP (1:500; ab53554; Abcam), anti-Iba1 (1:500; ab5076; Abcam), anti-8-OHdG (1:500; ab62623; Abcam), anti-c-fos (1:1000; 2250; Cell Signaling Technology, Beverly, USA), and anti-CGRP (1:1000; 14959; Cell Signaling Technology). Distal femur sections were incubated with anti-CD11b (1:500; GB11058; Servicebio, Wuhan, China) and anti-F4/80 (1:500; GB113373; Servicebio) antibodies. The sections were then washed 3 times with Tris-buffered saline and incubated with the following specific secondary antibodies for 2 h at room temperature: donkey anti-rabbit IgG (1:500; ab150073; Abcam), donkey anti-goat IgG (1:500; ab150132; Abcam), and donkey anti-mouse IgG (1:500; ab150108; Abcam). Following immunostaining procedures, the SC sections were examined using a laser scanning confocal microscope (FluoView FV1000; Olympus, Tokyo, Japan). For quantification of the number of immunopositive cells, 6 sections were randomly selected from each mouse. Cell counts were averaged to reflect the number of positive cells in the entire spinal dorsal horn. Fiji ImageJ software was used to quantify the colocalization of 8-OHdG with NeuN, GFAP or iba1 by calculating Pearson’s correlation coefficient, which estimates the degree of overlap between fluorescence signals obtained in two channels. The degree of colocalization according to the Pearson’s coefficient values was categorized as very strong (0.85 to 1.0), strong (0.49 to 0.84), moderate (0.1 to 0.48), or weak (‒0.26 to 0.09) based on previous reports [23].

### Quantitative polymerase chain reaction (qPCR)

Under deep anesthesia, the L4‒L5 spinal cord segments of the mice were quickly removed and analyzed. Total RNA was isolated with Trizol reagent (R401-01; Vazyme, Nanjing, China) according to the manufacturer’s instructions. cDNA was then synthesized using 5X PrimeScript RT Master Mix (TaKaRa, Dalian, China). Quantitative real-time polymerase chain reaction (TaKaRa) was performed in a 20-μL reaction mixture consisting of 10 μL of 2× SYBR Premis Ex TaqTM II, 2 μL of cDNA, 1 μL of forward primer, 1 μL of reverse primer and 6 μL of ddH2O. The thermal cycling conditions used were 95°C for 5 min, 40 cycles of 95°C for 5 s and 58°C for 30 s, and 72°C for 30 s. The specific primers used for detection are listed in Supplementary Table S1. Relative mRNA levels were calculated using the 2 ^‒ΔΔCT^ method [30]. Gene expression was normalized to that of the housekeeping control gene *GAPDH*.

### Western blot (WB) analysis

On the ipsilateral side, the L4‒L5 spinal cord segments were quickly removed from the deeply anesthetized mice and stored at –80°C. The tissues were homogenized in RIPA lysis buffer containing a protease inhibitor cocktail (Servicebio). The protein concentrations of the lysates were estimated using the BCA kit (23225; Thermo Scientific, Waltham, USA), and the total protein content between samples was equalized. Total protein was separated by SDS-PAGE and electrophoretically transferred onto polyvinylidene fluoride membranes (PVDF; IPV H00010; Millipore, Billerica, USA). The PVDF membranes were blocked with 5% fat-free milk (w/v) for 2 h and incubated with specific primary antibodies at 4°C overnight. The following primary antibodies were used: anti-AMPKα (1:1000; 5831; Cell Signaling Technology), anti-p-AMPKα (1:1000; 2535; Cell Signaling Technology), anti-P38 (1:1000; 8690; Cell Signaling Technology), anti-p-P38 (1:1000; 4511; Cell Signaling Technology), anti-JNK (1:1000; 9252; Cell Signaling Technology), anti-p-JNK (1:1000; 4668; Cell Signaling Technology), anti-ERK (1:1000; 4695; Cell Signaling Technology), anti-p-ERK (1:1000; 4370; Cell Signaling Technology), and anti-GAPDH (1:1000; GB12002; Servicebio). The membranes were then incubated with peroxidase-conjugated secondary antibodies (Servicebio). The membrane was visualized with enhanced chemiluminescence (ECL) substrate (3210; Thermo Scientific6) and exposed using a ChemiDoc XRS imaging system (Bio-Rad, Hercules, USA). The intensity of the blots was quantified by ImageJ software (National Institutes of Health, Bethesda, USA).

### Enzyme-Linked Immunoassay (ELISA)

Total proteins in plasma were taken from experimental mice, and MOTS-c in plasma was quantified using commercially available ELISA kits (CEX132Mu; Cloud-Clone Crop, Wuhan, China) according to the manufacturer’s instructions.

### Bone histology and tartrate-resistant acid phosphatase staining of mouse femurs

Mice were deeply anesthetized and intracardially perfused with 20 mL of PBS followed by 20 mL of 4% paraformaldehyde (PFA). The femora were removed and then fixed for 6–8 h in the same fixative at 4°C. After demineralization in EDTA (10%) for 10 days, the femur samples were dehydrated in an ascending gradient of ethanol (75%–100%) followed by paraffin embedding. Serial sections of trabecular bone were obtained from the distal femur at a thickness of 8 μm, followed by staining with hematoxylin and eosin (H&E) and tartrate-resistant acid phosphatase (TRAP) using TRAP kits (G1050-50; Servicebio). The images (H&E and TRAP) were captured with an ordinary optical microscope (ZEISS, Wetzlar, Germany). Static histomorphometric bone analyses for osteoclast number and osteoblast number were performed using ImageJ (NIH). Six sections were randomly chosen and used for quantification.

### *In vitro* experiments

BV2 microglia were cultured in DMEM (SH30022.01; HyClone, Carlsbad, USA) supplemented with 10% fetal bovine serum (A3160902; Gibco, Carlsbad, USA) and 1% penicillin and streptomycin (C0222; Beyotime, Shanghai, China) in an incubator at 37°C with 5% CO _2_. First, as described in a previous study [31], BV2 cells were treated with LPS at a concentration of 100 ng/mL. At the same time, different concentrations of MOTS-c (10 ^–5^, 10 ^–6^, 10 ^–7^, 10 ^–8^, and 10 ^–9^ M) were added to the experimental group. An equal volume of PBS was added to the control group. After 6, 12, and 24 h of incubation, all cells were collected. After RNA was extracted from the cells, the mRNA expressions of related inflammatory factor genes was detected by qPCR. After 24 h of LPS treatment, MOTS-c was added dropwise to the LPS-treated cells, and the vehicle-treated cells received an equal volume of PBS. After 6 h of MOTS-c treatment, all cells were harvested. All cell lysates were prepared for the evaluation of mitochondrial function. According to the manufacturer’s instructions, the intracellular ROS level was measured using DCFH-DA dye (Sigma), and the mitochondrial membrane potential was investigated using a JC-1 kit (C2003S; Beyotime).

### *In vitro* cytotoxicity assay

MOTS-c *in vitro* cytotoxicity was tested in Lewis lung cancer cells using a CCK8 cell proliferation assay kit (Beyotime). Cells were seeded in flatbottom 96-well plates at 5000 cells per well with 100 μL of medium per well. MOTS-c was added to each well at the desired final concentration (10 ^–5^, 10 ^–6^, 10 ^–7^, 10 ^–8^ and 10 ^–9^ M). After incubation for 6, 12 and 24 h at 37°C, 10 μL of CCK8 reagent was added to each well, and the plate was incubated at 37°C for another 2 h. At the end of the incubation, the absorbance at 490 nm was measured on a Flex Station three plate reader (Molecular Devices, San Jose, USA).

### Transmission electron microscopy

Two weeks after BCP, the mice underwent heart perfusion with 4% paraformaldehyde after deep anesthesia and saline perfusion. Lumbar cord tissue (L4–L5) was isolated, excised, cut to 1 mm× 3 mm, fixed in 2.5% buffered glutaraldehyde and 1% osmium tetroxide (Servicebio) for 48 h, and then dehydrated through an alcohol gradient and acetone before the tissue was embedded in resin. The tissue was sliced into 0.05 μm-thick sections using Pathology Slicer (Leica, Wetzlar, Germany). Finally, sections from each group were imaged using a transmission electron microscope (HT7700; Hitachi, Tokyo, Japan) after contrast staining with uranyl acetate and lead citrate.

### Statistical analysis

The statistical software SPSS 19.0 (IBM Corp., Armonk, USA) was used for statistical analysis of the data. All the data are presented as the mean±standard error of mean (mean±SEM). Comparisons of data between different groups were conducted using one-way ANOVA followed by Bonferroni post hoc correction. Comparisons of time-series data were performed with two-way repeated-measures ANOVA. *P*<0.05 was considered to indicate statistical significance.

## Results

### Chronic injection of MOTS-c attenuates mechanical allodynia in mice with BCP

**Figure 1:**
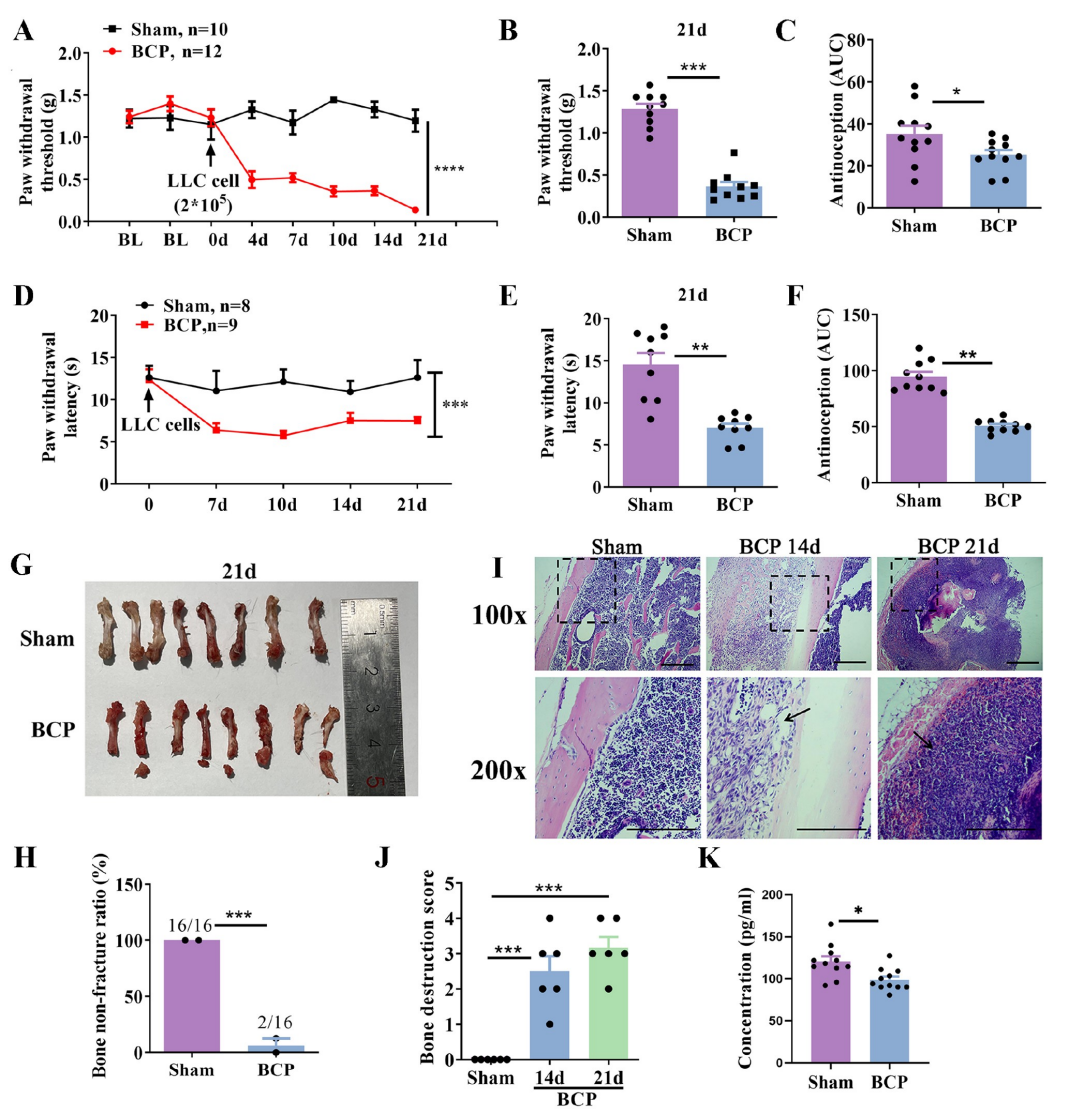
Figure 1 Bone cancer pain models were established by inoculation of Lewis lung cancer cells into the medullary cavity of the distal left femur (A,D) Mechanical allodynia and thermal allodynia were investigated prior to surgery and on days 4, 7, 10, 14 and 21 after LLC inoculation ( n=10–12). (B,E) Changes in mechanical allodynia and thermal allodynia on day 21 after LLC cell inoculation ( n=10–12). (C,F) The extent and duration of analgesia were estimated by the area under the curve (AUC) of the PWT vs time (0–21 days). (G) Representative images of LLC-induced femur fracture in mice on day 21. (H) Ratio of LLC-induced femur fracture in mice on day 21. (I) Representative images of H&E-stained femurs from mice on days 14 and 21. Scale bar: 100 μm. (J) H&E staining score ( n=6). (K) The levels of MOTS-c in plasma were examined by ELISA ( n=11). Data are expressed as the mean±SEM. * P<0.05, ** P<0.01 and *** P<0.001 compared with the sham group.

**Figure 2:**
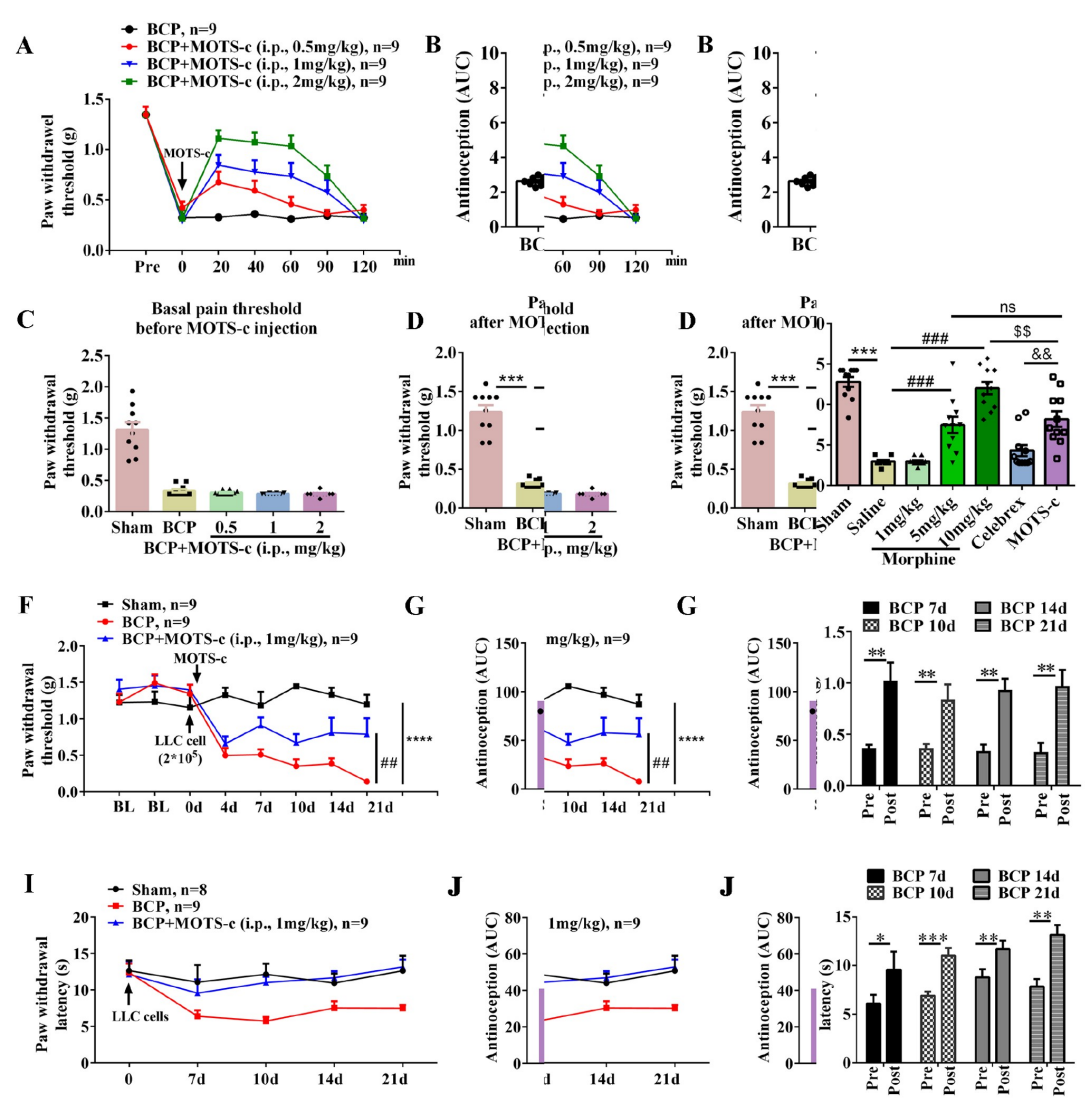
Figure 2 Chronic MOTS-c treatment attenuated LLC-induced mechanical pain and thermal hypersensitivity (A) Mechanical allodynia was investigated before the i.p. administration of MOTS-c (0.5, 1, or 2 mg/kg) to LLC-induced BCP mice ( n=10). (B) The extent and duration of analgesia were estimated by the area under the curve (AUC) of the PWT vs time (0–120 min). (C,D) Dose-response effects of MOTS-c (0.5, 1, and 2 mg/kg) 30 min after i.p. administration in LLC-induced BCP mice ( n=9). (E) The effects of morphine (1, 5, and 10 mg/kg), celebrex (30 mg/kg), and MOTS-c (1 mg/kg) on LLC-induced BCP mice ( n=10). (F) Mechanical allodynia was investigated prior to surgery and on days 4, 7, 10, 14 and 21 after LLC cell inoculation ( n=9). (G,J) The extent and duration of analgesia were estimated by the area under the curve (AUC) of the PWT vs time (0–21 days). (I) Thermal allodynia was investigated prior to surgery and on days 7, 10, 14 and 21 after LLC cell inoculation ( n=8–9). (H,K) The effects of pre- and post-administration of MOTS-c on the PWT and PWL were investigated on days 7, 10, 14, and 21 ( n=9). Data are expressed as the mean±SEM. ns indicates no significance. * P<0.05, ** P<0.01, *** P<0.001, and **** P<0.0001 compared with preadministration. ** P<0.01 and *** P<0.001 compared with the sham group. # P<0.05, ## P<0.01, ### P<0.001 compared with the BCP group. $$ P<0.01 compared with the BCP+morphine (10 mg/kg) group. && P<0.01 compared with the BCP+celebrex (30 mg/kg) group.

To determine whether systemic administration of MOTS-c could provide long-term therapeutic effects against BCP, a syngeneic murine bone cancer pain model was established by inoculating LLC cells (2×10 ^5^ cells in 10 μL) into the femora of C57BL/6 mice. To verify the establishment of the BCP model, H&E staining and behavioral tests were performed. As shown in Figure 1, BCP mice exhibited a marked decrease in the PWT after 4 days, and this effect lasted more than 20 days after tumor implantation. Pain-related behavior was evaluated using the von Frey test and the thermal pain test for mechanical allodynia and thermal allodynia. On day 21 (d21) after LLC inoculation, the mechanical and thermal withdrawal thresholds of the paw were significantly lower than those of the sham group ( Figure 1B,E). In addition, the mice were euthanized, and the tumor-bearing femurs were collected. None (0/16) of the sham mice developed bone fractures, whereas 87.5% (14/16) of the BCP mice developed distal bone fractures ( Figure 1G,H). H&E staining revealed infiltration of bone marrow spaces by a malignant tumor ( Figure 1I). Compared with those in the sham group, cancer-induced bone loss and lesions were more evident at d21 after LLC cell inoculation ( Figure 1I,J). We observed that the plasma level of endogenous MOTS-c in the BCP group was significantly lower than that in the sham group ( Figure 1K). We further examined whether i.p. injection of MOTS-c modulates the mechanical pain threshold in BCP mice. As shown in Figure 2A,B, i.p. injection of MOTS-c (0.5, 1 or 2 mg/kg) significantly reversed mechanical allodynia ( *P*<0.01 for 0.5 mg/kg; *P*<0.001 for 1 or 2 mg/kg) ( Figure 2A,B). The AUCs were calculated at 0–120 min from the MOTS-c dose-response curve using trapezoidal rules and were analyzed using one-way ANOVA followed by Bonferroni *post hoc* correction. The extent and duration of analgesia were estimated according to the AUC values (0–120 min) ( Figure 2B). MOTS-c had a pronounced dose-response relationship at 30 min after injection ( Figure 2C,D). Moreover, we used two positive controls, a nonsteroidal anti-inflammatory drug (celebrex, i.p., 30 mg/kg) and an opioid analgesic for BCP (morphine, i.p., 1, 5, and 10 mg/kg). The analgesic effects of MOTS-c (1 mg/kg, i.p.) were greater than those of celebrex (30 mg/kg, i.p.) and were not significantly different from those of morphine (5 mg/kg, i.p.). MOTS-c produced antinociceptive effects similar to those of celecoxib and morphine in BCP ( Figure 2E). Subsequently, consecutive i.p. administration of MOTS-c on postoperative days 7, 10, 14, and 21 reversed mechanical allodynia and thermal hyperalgesia in BCP mice ( *P*<0.01 for the BCP group vs 1 mg/kg MOTS-c+BCP group; *P*<0.001 for the BCP group vs 2 mg/kg MOTS-c+BCP group; Figure 2F–K). Taken together, these results indicate that MOTS-c treatment induced dose-dependent antinociceptive effects in mice with BCP.

### MOTS-c treatment protects against cancer-induced bone pain and bone destruction

**Figure 3:**
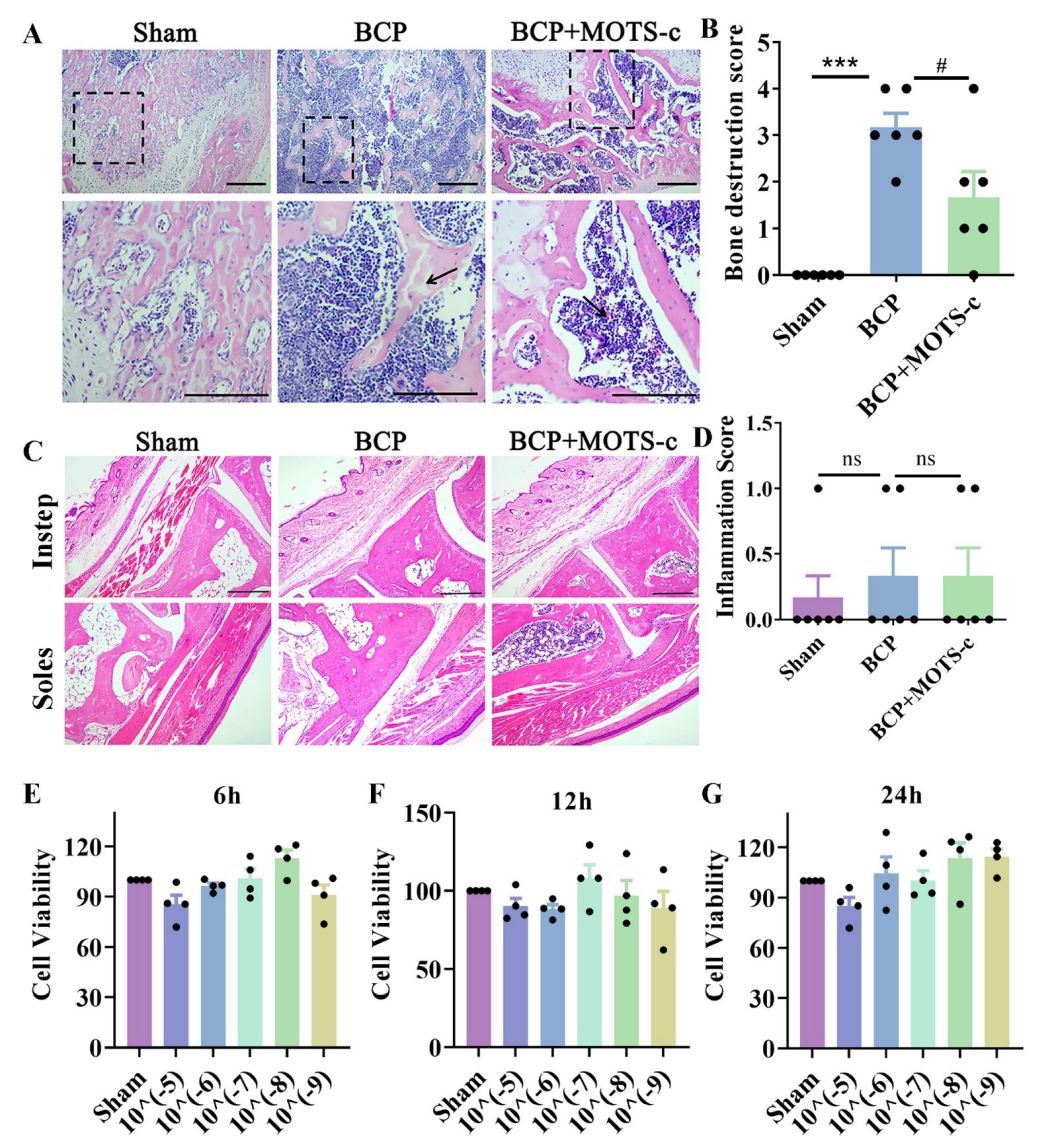
Figure 3 Chronic MOTS-c treatment protected against cancer-induced bone destruction but had no effect on cancer cell proliferation (A) Representative images of H&E-stained distal femurs of mice on day 14. Scale bar: 100 μm. (B) Quantitative bone destruction score analysis of the different groups ( n=6). (C) Representative images of H&E staining of the surface structure of hindpaw skin. Scale bar: 100 μm. (D) Quantitative inflammation score analysis of the different groups ( n=6). (E) Cell viability was determined by the CCK8 assay. MOTS-c treatment (10 ‒9 to 10 ‒5 M) did not change cancer cell proliferation at multiple time points (6, 12, and 24 h). Data are expressed as the mean±SEM. ns indicates no significance. *** P<0.001 compared with the sham group. # P<0.05 compared with the BCP group.

To examine the role of MOTS-c in BCP in peripheral bone tissues, an H&E staining assay was performed. Histological analysis revealed that compared with those in the sham group, there was increased infiltration of cancer cells into the bone marrow spaces and a loss of normal bone structure ( *P*<0.001 for the sham group vs the BCP group; Figure 3A,B), whereas bone loss was attenuated after MOTS-c application ( *P*<0.05 for the BCP group vs the BCP+MOTS-c group; Figure 3A,B). In addition, CCK-8 assay was performed to confirm the effects of MOTS-c on cancer cell proliferation. MOTS-c (10 ^–9^–10 ^–5^ M) had no effect on cancer cell proliferation at multiple time points compared with that in the sham group ( Figure 3E–G).

Given that a large number of cancer cells evoke the infiltration of immune cells such as granulocytes and macrophages [ 2, 5, 8] , the von Frey behavioral test was performed using the hind paws of mice with BCP. Thus, we also examined inflammatory infiltration in the hind paw. HE staining revealed that there were no significant differences among the sham, BCP and MOTS-c treatment groups ( Figure 3C,D).

### MOTS-c promotes the OPG/RANKL ratio and inhibits cancer-induced osteoclastogenesis in mice with BCP

**Figure 4:**
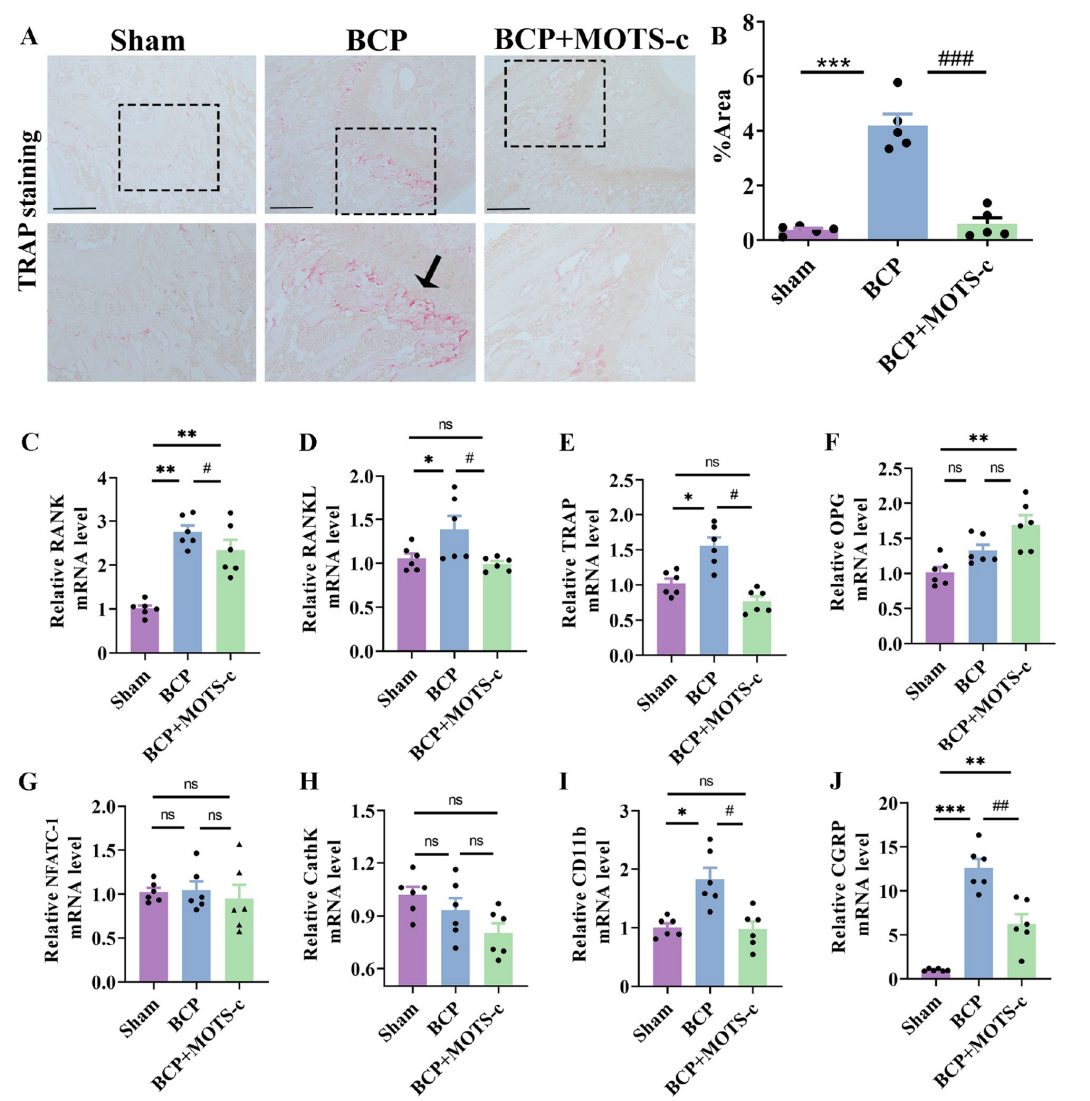
Figure 4 Chronic administration of MOTS-c inhibited cancer-induced osteoclastogenesis in mice with BCP (A) Representative images of TRAP-stained distal femurs of mice in different groups on day 14. Scale bar: 100 μm. (B) Quantitative analysis of the osteoclast area (arrow) in the different groups ( n=5). (C‒J) The mRNA expressions of RANK, RANKL, TRAP, OPG, NFATC-1 and CathK were measured in sham, BCP, and BCP+MOTS-c mice by qPCR ( n=6). Data are expressed as the mean±SEM. ns indicates no significance. * P<0.05, ** P<0.01 and *** P<0.001 compared with the sham group. # P<0.05, ## P<0.01 and ### P<0.001 compared with the BCP group.

As our data suggested that MOTS-c could reduce bone destruction, we then investigated whether the bone-protective effects are mediated by direct effects on osteoclastogenesis, and TRAP staining was performed to measure the number of osteoclast cells [9]. TRAP staining revealed that sham mice exhibited significantly fewer osteoclasts ( Figure 4A), whereas TRAP-positive osteoclasts were significantly more abundant in the tumor-bearing distal femora of BCP mice ( *P*<0.001 for the sham group vs the BCP group; Figure 4A,B). Moreover, MOTS-c treatment (1 mg/kg, i.p.) significantly inhibited osteoclast formation in BCP mice ( *P*<0.001 for the BCP group vs the BCP+MOTS-c group; Figure 4A,B).

According to recent reports [ 32, 33] , osteocytes secrete OPG and RANKL to regulate osteoclastogenesis. Thus, we examined the effect of MOTS-c on the mRNA expressions of osteoclastogenesis-related factors such as *RANK*, *RANKL*, *TRAP*, *OPG*, *NFATC-1* and *CathK*. Consistent with the TRAP staining results, the expressions of osteoclast molecular markers ( *RANK*, *RANKL*, and *TRAP*) were inhibited by MOTS-c treatment ( *P*<0.05 for *RANK*, *RANKL* and *TRAP*; Figure 4C–J). A significant increase in *OPG* expression was observed in the MOTS-c-treated group ( *P*<0.05 for *OPG*; Figure 4F).

### MOTS-c inhibits the activation of peripheral macrophages and CGRP-expressing sensory neurons in local bone tissues

**Figure 5:**
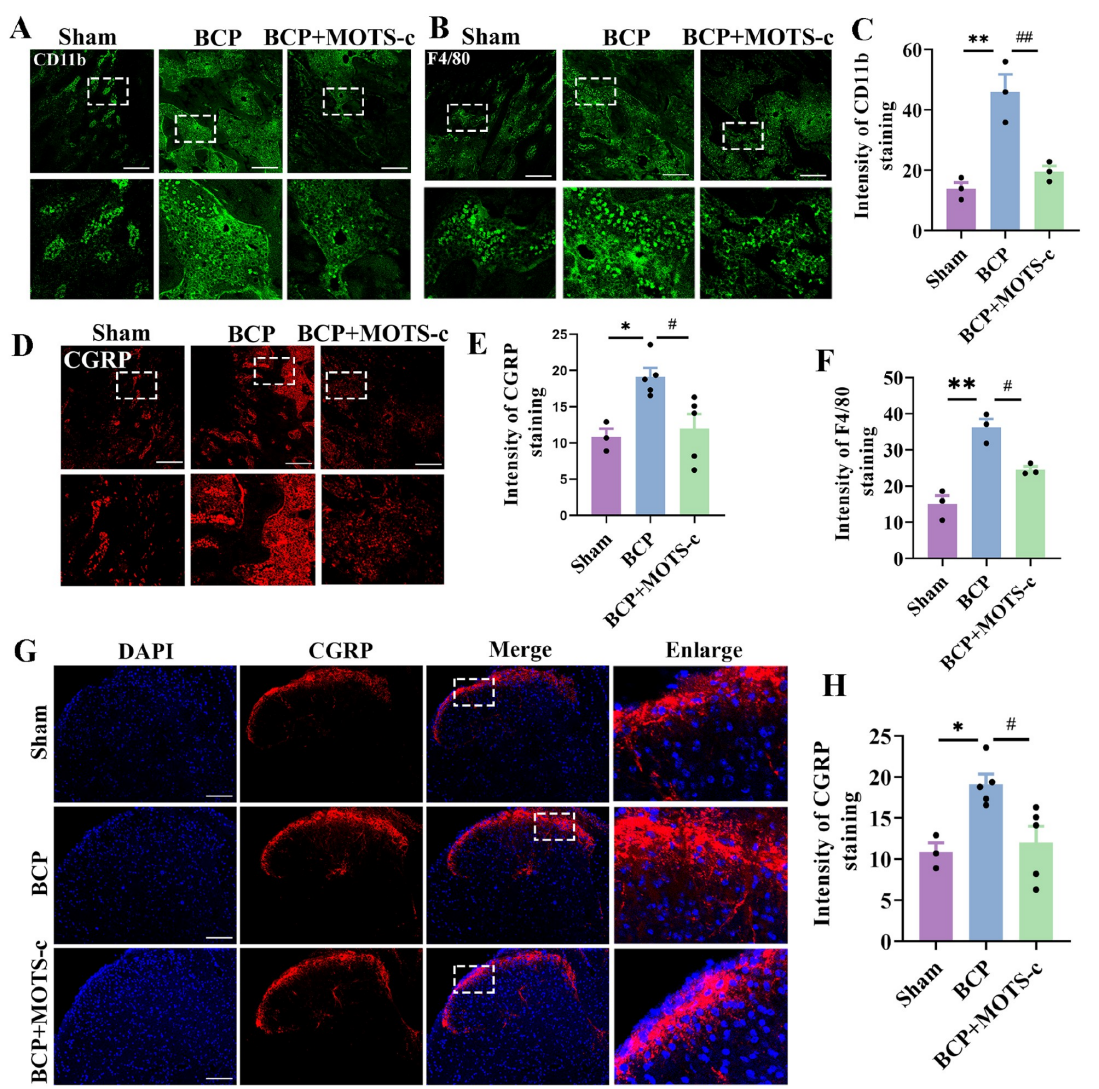
Figure 5 Chronic administration of MOTS-c inhibited the activation of peripheral macrophages and CGRP-expressing sensory neurons (A,B,D) Representative images of CD11b-positive (green), F4/80-positive (red) and CGRP-positive (red) cells in the different groups are shown. Scale bar: 100 μm. (C,E,F) Immunofluorescence analysis of the protein expressions of CD11b, F4/80, and CGRP in the distal femur bone tissues of mice ( n=3–5). Tissues were collected on day 14 after MOTS-c treatment. (G) Representative images of CGRP immunofluorescence (red) in the spinal dorsal horn. Scale bar: 100 μm. (H) Qualitative data showing the intensity of CGRP positivity in the spinal dorsal horn ( n=5). Data are expressed as the mean±SEM. ns indicates no significance. * P<0.05, ** P<0.01 compared with the sham group. # P<0.05, ## P<0.01 compared with the BCP group.

A large number of proliferating cancer cells and activated osteoclasts evoke the infiltration of immune cells, especially macrophages [ 34, 35] . Thus, we investigated whether macrophages are involved in the therapeutic effects of MOTS-c. The expressions of CD11b and F4/80, which are macrophage markers, were significantly greater in the BCP group than in the sham group ( *P*<0.01 for the sham group vs the BCP group; Figure 5A–C). However, MOTS-c treatment markedly inhibited the expressions of CD11b and F4/80 in the tumor-bearing distal femora of BCP mice to a level comparable to that in BCP mice ( *P<*0.01 for CD11b, *P*<0.05 for F4/80; Figure 5A,B,C,F).

Numerous studies have demonstrated that tissue injury and local stimulation induce the excitation of peripheral sensory neurons [ 36, 37] . Thus, we also investigated the effect of MOTS-c on peripheral and central innervation patterns, such as CGRP sensory neurons. Notably, compared with the sham group, the BCP group exhibited significantly greater peripheral or central innervation density ( *P*<0.05 for the sham group vs the BCP group; Figure 5D,G), although MOTS-c treatment resulted in significant downregulation of CGRP expression in peripheral and central innervation patterns ( *P*<0.05 for the BCP group vs the BCP+MOTS-c group; Figure 5D,H).

### MOTS-c improves BCP-induced mitochondrial dysfunction in mice with BCP

**Figure 6:**
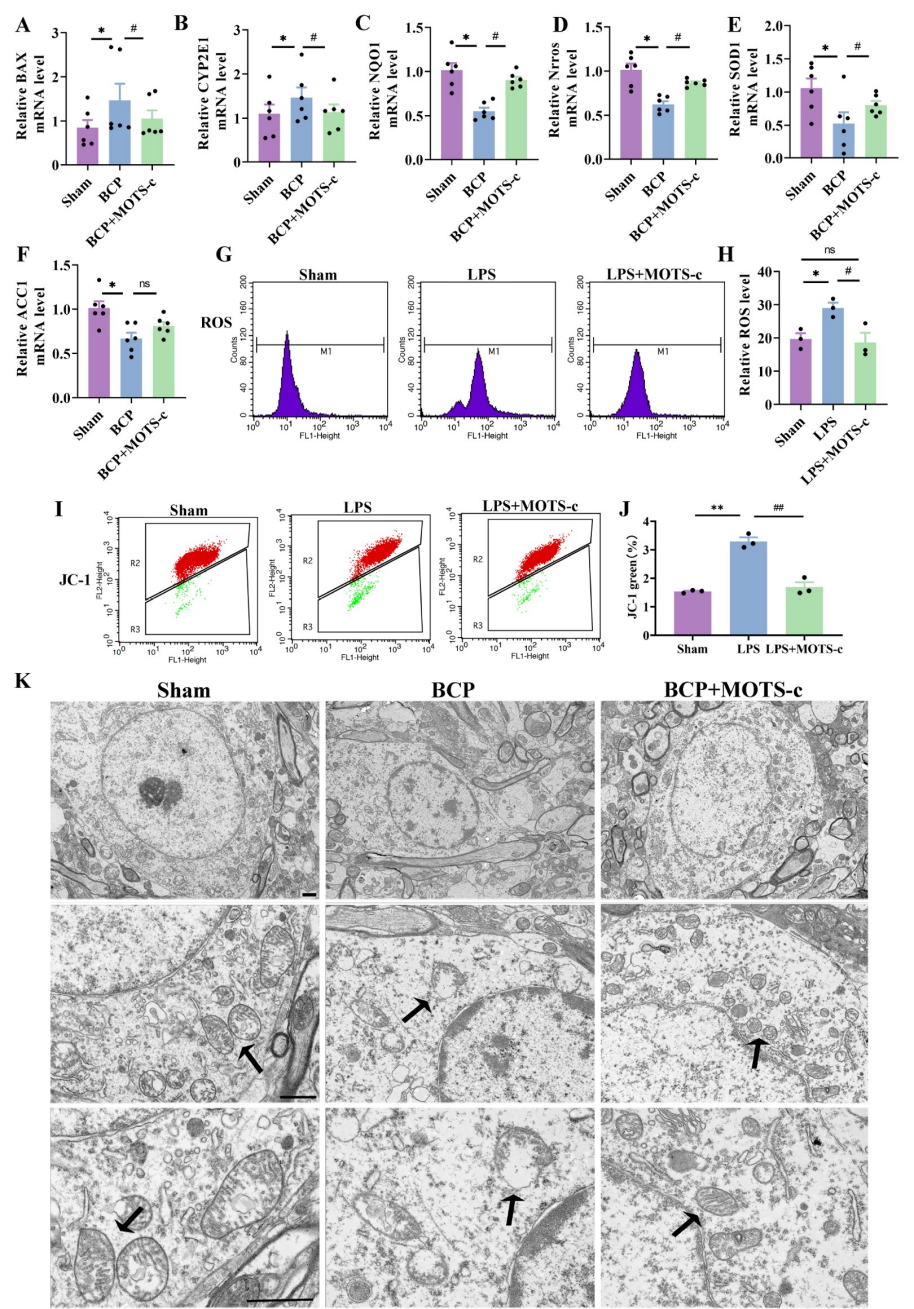
Figure 6 MOTS-c improved BCP-induced mitochondrial dysfunction in mice with BCP (A‒F) The mRNA expressions of BAX, CYP2E1, NQO1, Nrros, SOD1, and ACC1 in the spinal cord were measured by qPCR ( n=6). (G,H) The level of intracellular ROS was determined using the fluorescent dye DCFH-DA and quantified by flow cytometry ( n=3). (I,J) The level of intracellular JC-1 was determined and quantified by flow cytometry ( n=3). (K) Representative transmission electron microscopy image of mitochondria (arrows) in neurons in the spinal dorsal horn. Scale bar: 1 μm. Data are expressed as the mean±SEM. ns indicates no significance. * P<0.05, ** P<0.01 compared with the sham group. # P<0.05, ## P<0.01 compared with the BCP group.

MOTS-c originates from the mitochondrial genome, and mitochondrial dysfunction has been reported to be involved in the development of BCP [ 10, 38, 39] . Thus, we investigated whether the mitochondrial axis is involved in the effects of MOTS-c on BCP. In the spinal cord of mice with BCP, the mRNA expressions of markers related to mitochondrial dysfunction ( *CYP2E1* and *Bax*) were inhibited by MOTS-c treatment ( *P*<0.05 for *Bax* and *CYP2E1*; Figure 6A,B). Furthermore, MOTS-c markedly increased the expressions of factors associated with improved mitochondrial function ( *NQO1*, *Nrros*, *SOD1*, and *ACC1*) at the mRNA levels in mice with BCP ( *P*<0.05 for *NQO1*, *Nrros*, *SOD1*, and *ACC1*; Figure 6C–F).

Moreover, we examined the effects of MOTS-c on ROS production and mitochondrial membrane potential using DCFH-DA and JC-1 molecular probe assays. MOTS-c treatment (10 ^–5^ M) significantly inhibited LPS-induced ROS production *in vitro* ( *P*<0.05 for the LPS group vs the LPS+MOTS-c group; Figure 6G,H). As shown in Figure 6I,J, the JC-1 assay showed that LPS treatment significantly enhanced JC-1 ( *P*<0.01 for the sham group vs the LPS group), whereas MOTS-c treatment (10 ^–5^ M) markedly decreased the level of JC-1 ( *P*<0.05 for the LPS group vs the LPS+MOTS-c group). Furthermore, transmission electron microscopy was performed to observe mitochondrial morphology. The results revealed that mitochondrial cristae were lost and that the size and perimeter of mitochondria were decreased in BCP mice. Similar to those in the sham group, the mitochondria in the MOTS-c treatment group contained a complete inner membrane, an outer membrane, and oval-shaped cristae ( Figure 6K).

### MOTS-c inhibits ROS-induced excess oxidative damage in neurons and attenuates neuronal activation in mice with BCP

**Figure 7:**
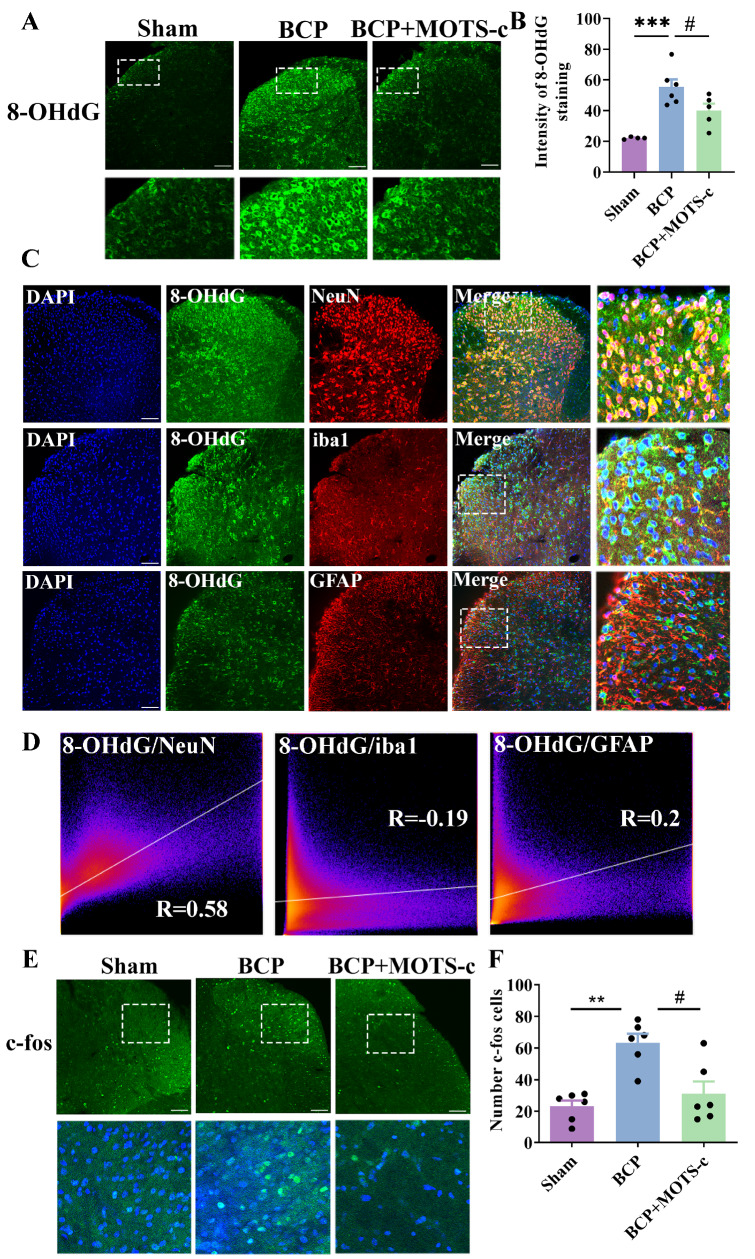
Figure 7 MOTS-c attenuated excess ROS-induced oxidative damage in neurons and inhibited c-fos activation in mice with BCP (A) The expression of 8-OHdG in the spinal dorsal horn. Scale bar: 100 μm. Tissues were collected on day 14 after MOTS-c treatment. (B) The qualitative data showing the intensity of 8-OHdG in the spinal dorsal horn ( n=6). (C) Immunofluorescence showing the colocalization of 8-OHdG (green) with neurons (NeuN, red), microglia (iba1, red), and astrocytes (GFAP, red) in the spinal dorsal horn. Scale bar: 100 μm. (D) It shows the analysis of colocalization in different groups ( n=6). (E) Representative images of c-fos immunofluorescence (green) in the spinal dorsal horn (scale bar 100=μm). (F) The qualitative data show the number of c-fos-positive neurons in the spinal dorsal horn ( n=6). Tissues were collected on day 14 after MOTS-c treatment. Data are expressed as the mean±SEM. ** P<0.01, *** P<0.001 compared with the sham group. # P<0.05 compared with the BCP group.

8-Hydroxydeoxyguanosine (8-OHdG) is widely used as a biomarker of ROS-induced endogenous DNA oxidative damage [40]. As shown in Figure 7A,B, the level of 8-OHdG in the spinal cord of BCP mice increased significantly ( *P*<0.001 for the sham group vs the BCP group), whereas MOTS-c administration (1 mg/kg, i.p.) resulted in a significant decrease in 8-OHdG immunoreactivity ( *P*<0.05 for the BCP group vs the BCP+MOTS-c group; Figure 7A,B). Next, analysis of the cellular distribution revealed that ROS-induced DNA oxidative damage was distributed throughout the cells in the spinal dorsal horn. Confocal images showed that 8-OHdG immunoreactivity primarily colocalized with neurons (as indicated by NeuN, a neuronal marker) but not with astrocytes (GFAP, an astrocyte marker; Figure 7C). Although 8-OHdG colocalized primarily with neurons, a notable amount of 8-OHdG was found to colocalize with microglia (with iba1 as a microglial marker).

Furthermore, the expression of c-fos has been used as an indicator of neuronal activity. The expression of c-fos increased in the spinal cord of BCP mice ( *P*<0.01 for the sham group vs the BCP group), whereas MOTS-c (1 mg/kg, i.p., daily for 21 days) negatively regulated the expression of c-fos ( *P*<0.05 for the BCP group vs the BCP+MOTS-c group) ( Figure 4D‒F).

### MOTS-c suppresses microglial activation and proinflammatory cytokine production in the spinal cords of mice with BCP

**Figure 8:**
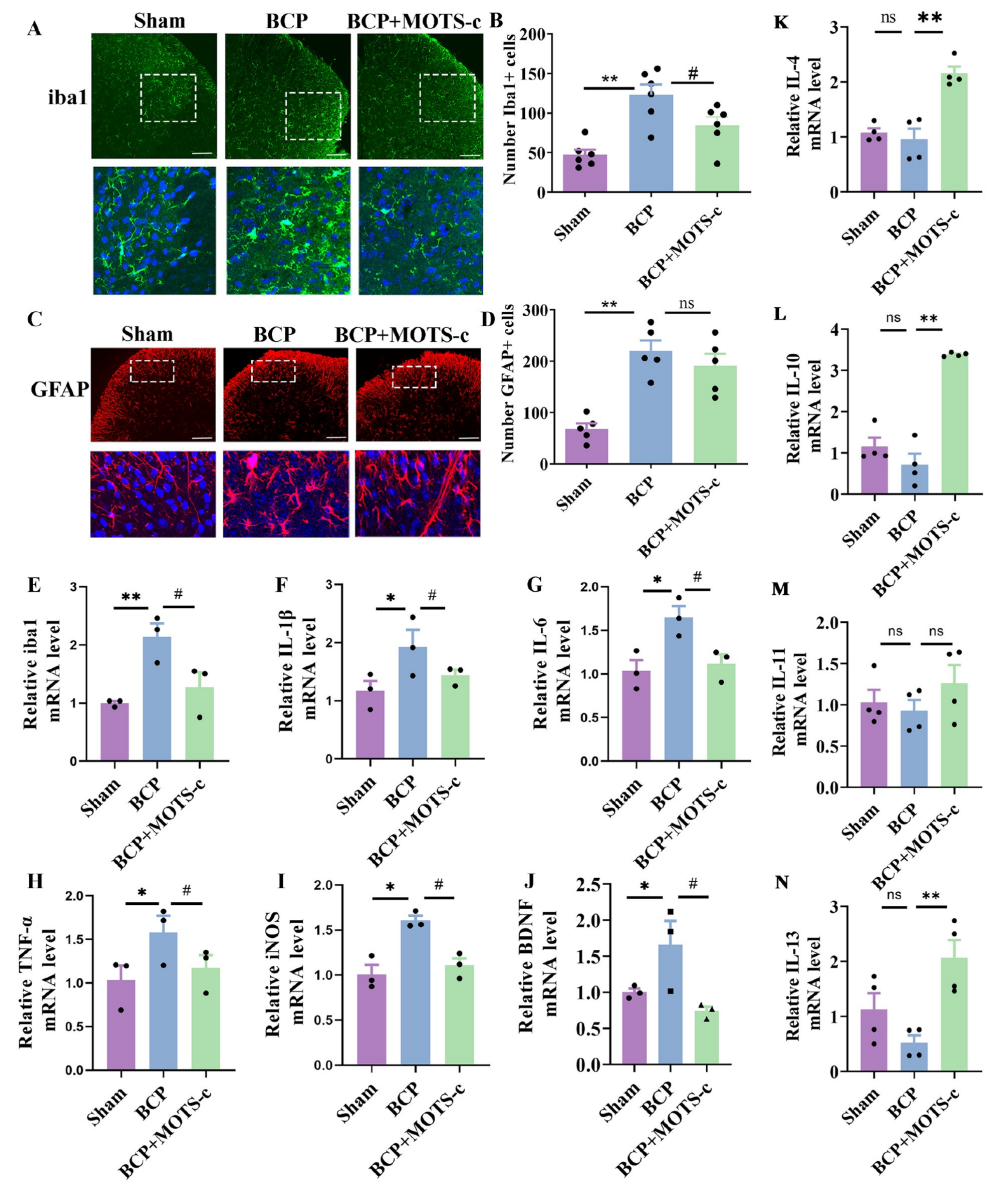
Figure 8 MOTS-c suppressed the activation of microglia and proinflammatory cytokine production in the spinal cords of mice with BCP (A,C) Representative images of iba1 immunofluorescence (green) and GFAP immunofluorescence (red) in the dorsal horn. Scale bar: 100 μm. (B,D) Qualitative data showing the number of positive microglia and astrocytes in the spinal dorsal horn ( n=5‒6). Tissues were collected on day 14 after MOTS-c treatment. (E‒J) The mRNA expressions of iba1, IL-1β, IL-6, TNF-α, iNOS and BDNF in the spinal cord of sham, BCP, and BCP+MOTS-c mice were measured by qPCR ( n=3). (K‒N) The mRNA expressions of IL-4, IL-10, IL-11 and IL-13 in the spinal cord of sham, BCP, and BCP+MOTS-c mice were measured by qPCR ( n=3). Data are expressed as the mean±SEM. ns indicates no significance. * P<0.05, ** P<0.01 compared with the sham group. # P<0.05 compared with the BCP group.

Given that astrocytes and microglia have been recognized as important contributors to the development of BCP [ 41, 42] , we also tested whether astrocytes and microglia are involved in the therapeutic effects of MOTS-c. Iba1 and GFAP were used as markers of microglia and astrocyte activity, respectively. As shown in Figure 8A–D, compared with the sham group, the BCP group exhibited a significant increase in iba1-positive microglia ( *P*<0.01 for the sham group vs the BCP group) and GFAP-positive astrocytes ( *P*<0.01 for the sham group vs the BCP group). However, MOTS-c (1 mg/kg, ip, daily for 20 days) negatively regulated the expression of the marker iba1 ( *P*<0.05 for the BCP group vs the BCP+MOTS-c group) ( Figure 8A–D).

Activated microglia are considered the main source of proinflammatory cytokines [43], and we investigated the mRNA expressions of proinflammatory cytokines, including *iba1*, *TNF-α*, *IL-1β*, *IL-6*, *BDNF* and *iNOS*, and anti-inflammatory cytokines, including *IL-4*, *IL-10*, *IL-11* and *IL-13*. MOTS-c attenuated BCP-induced increases in the mRNA expressions of proinflammatory cytokines such as *iba1*, *TNF-α*, *IL-1β*, *IL-6*, *BDNF* and *iNOS* ( *P*<0.05 for *iba1*, *TNF-α*, *IL-1β*, *IL-6*, *BDNF* and *iNOS*; Figure 8E–J) and upregulated the expressions of anti-inflammatory cytokines such as *IL-4*, *IL-10* and *IL-13* ( Figure 8K‒N). Similar results were obtained in *in vivo* experiments ( Supplementary Figure S1).

### Spinal AMPK activation is required for the effects of MOTS-c in mice with BCP

**Figure 9:**
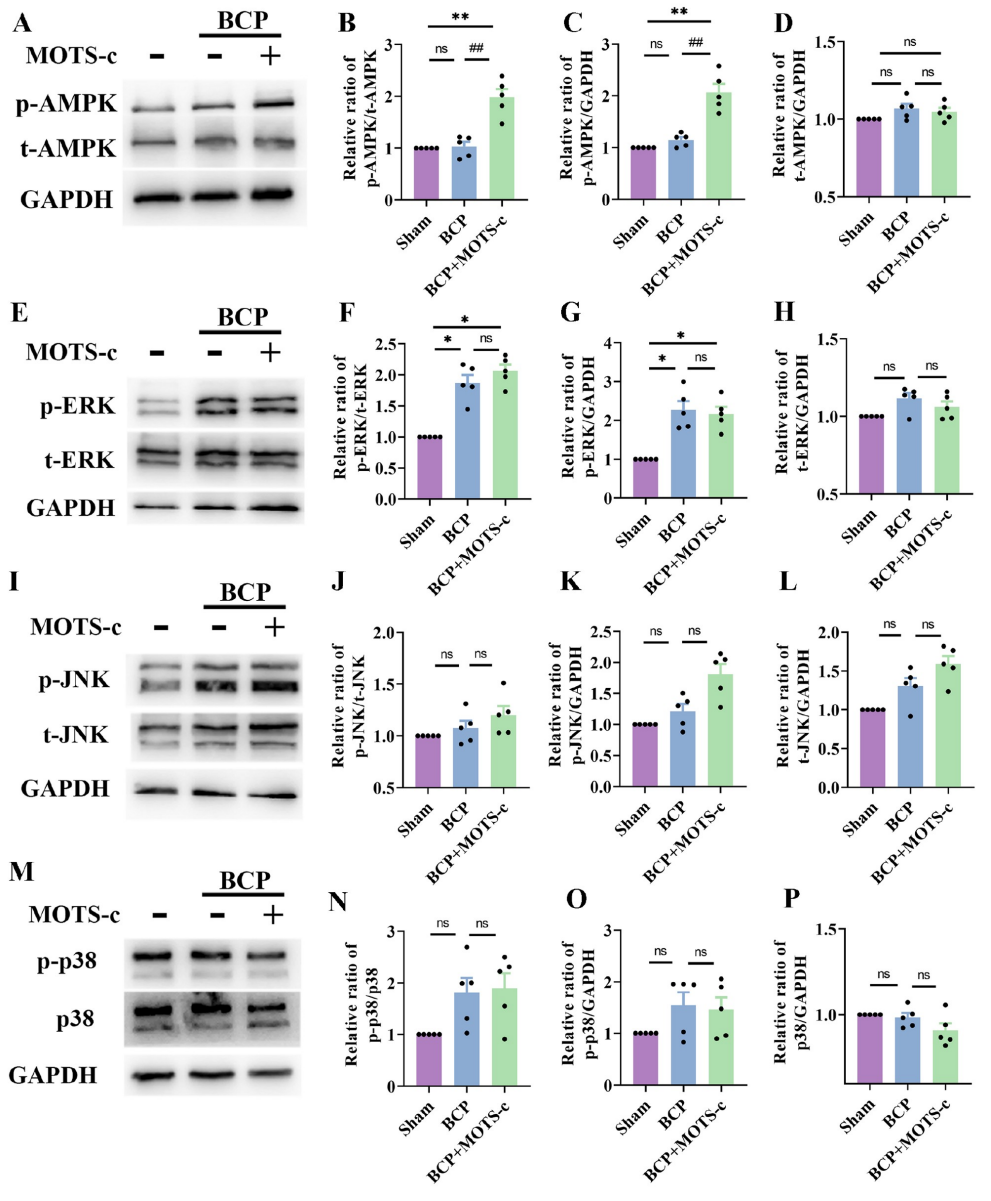
Figure 9 Chronic MOTS-c treatment upregulated p-AMPK expression but had no effect on ERK/JNK/p38 signalling (A) Western blot analysis of p-AMPK and t-AMPK in the different groups. (B‒D) The qualitative data showing the protein expression levels of p-AMPK and t-AMPK in the different groups ( n=5). (E) Western blot analysis of p-ERK and t-ERK in the different groups. (F‒H) Quantification of p-ERK and t-ERK protein expressions in the different groups ( n=5). (I) Western blot analysis of p-JNK and t-JNK in the different groups. (J-L) Quantification of p-JNK and t-JNK protein expressions in the different groups ( n=5). (M) Western blot analysis of p-p38 and t-p38 in the different groups. (N‒P) Quantification of p-p38 and t-p38 protein expressions in the different groups ( n=5). Data are expressed as the mean±SEM. ns indicates no significance. * P<0.05, ** P<0.01 compared with the sham group. ## P<0.01 compared with the BCP group.

The molecular mechanism of MOTS-c in the regulation of glucose metabolism, obesity, and other diseases is related to the AMPK and ERK signaling pathways, which in turn are essential for BCP [ 10, 14, 15, 23] . Therefore, we investigated the MAPK signaling pathway (p38, ERK and JNK) and the AMPK signaling pathway. As shown in Figure 9A–D, compared with the BCP group, MOTS-c treatment significantly increased the level of phosphorylated AMPK ( *P*<0.01 for the BCP group vs the BCP+MOTS-c group; Figure 9A–D). Conversely, total AMPK was not affected by MOTS-c treatment ( Figure 9D), which is in line with the results obtained with the use of behavioral antagonists. Compound C, an inhibitor of the AMPK pathway [44], was used to evaluate the effects of MOTS-c. As shown in Supplementary Figure S2, pretreatment with compound C (10 mg/kg, i.p.), 30 min before i.p. injection of MOTS-c (1 mg/kg), significantly blocked the effects of MOTS-c in BCP mice ( *P*<0.01 for the BCP+MOTS-c group vs BCP+compound C+MOTS-c group).

In addition to the AMPK signaling pathway, we investigated the classical inflammatory signaling pathway (the MAPK signaling pathway, which includes ERK, JNK, and p38 signaling), which is essential for the development of BCP. The levels of p38, ERK and JNK phosphorylation were significantly greater in the spinal cords of the mice with BCP than in those of the sham group ( *P*<0.05 for p-p38, p-JNK and p-ERK; Figure 9E–P). However, MOTS-c treatment did not alter the BCP-induced increase in phosphorylated p38, ERK or JNK expressions ( Figure 9E–P).

## Discussion

Current therapies for BCP are only partially beneficial [ 1, 6] . For this reason, the development of novel and more effective treatments is a current medical necessity. This study identified a unique and synergistic advantage of MOTS-c as a therapeutic strategy for the treatment of BCP. MOTS-c, a recently discovered mitochondrial-derived peptide, has been reported to be involved in regulating diabetes, insulin resistance, inflammation, aging, and osteoporosis [ 11– 13, 15] . Here, our study demonstrated that chronic intraperitoneal administration of MOTS-c robustly attenuated bone cancer-induced pain, indicating the potential pharmacological effects of MOTS-c peptide as a therapeutic agent against cancer-induced pain.

To date, we established a syngeneic murine BCP model by inoculating LLC cells into the femora of mice. In our intrafemoral inoculation model of bone cancer, inoculation of tumor cells led to progressive destruction of cortical bone, increased bone resorption, mechanical allodynia, and thermal allodynia. Notably, MOTS-c treatment directly suppressed osteoclast differentiation and attenuated mechanical allodynia and thermal hyperalgesia in mice with BCP. The antinociceptive effect of MOTS-c in BCP is likely particularly protective against bone cancer-induced osteoclastogenesis and bone destruction. This result is consistent with previous studies in which MOTS-c inhibited osteolysis in mouse calvaria by affecting osteocyte-osteoclast crosstalk and inhibiting inflammation [ 15, 17, 18] . However, our study focused on whether the effects of MOTS-c on BCP are related to a reduction in tumor burden. Further experiments revealed that MOTS-c has no effect on cancer cell proliferation at multiple time points or at multiple doses, suggesting that the persistent protective effects of MOTS-c on BCP are not associated with reduced tumor burden.

Our data revealed that MOTS-c treatment significantly inhibited LPS-induced ROS production *in vitro*. MOTS-c is a mitochondrial-derived peptide [10], whereas excess ROS-induced mitochondrial dysfunction is involved in the development of BCP [ 38, 45] . Thus, we investigated whether the mitochondrial axis is involved in the effects of MOTS-c on BCP. The results showed that the mRNA expressions of mitochondrial function-related markers ( *CYP2E1*, *Bax*, *SOD1*, *NQO1*, and *ACC1*) were improved with MOTS-c treatment both *in vivo* and *in vitro*. In addition, 8-OHdG is widely used as a biomarker of endogenous DNA oxidative damage induced by ROS [40]. In our experiments, MOTS-c treatment clearly attenuated ROS-induced DNA/RNA oxidative damage in the spinal dorsal horn. Moreover, double immunofluorescence staining revealed that 8-OHdG colocalized predominantly with spinal neurons rather than with microglia or astrocytes, suggesting that the effect of MOTS-c on spinal neurons may be more important for BCP treatment. Furthermore, the expression of c-fos, an indicator of neuronal activity, was downregulated by MOTS-c in the spinal cords of mice with BCP. The effects on c-fos were consistent with the colocalization of 8-OHdG with neurons. Thus, MOTS-c inhibited excess ROS-induced oxidative damage in neurons and attenuated neuronal activation in mice with BCP.

Growing evidence has illustrated that oxidative stress also stimulates the inflammatory response, which induces the expressions of various genes, such as genes encoding inflammatory factors and cytokines [ 46, 47] . Furthermore, an excess of proinflammatory cytokines can act as ROS-activating factors and promote oxygen radical production [ 46, 47] . Moreover, astrocytes and microglia have been reported to play important roles in the development of chronic pain [ 43, 48] . Importantly, our data showed that MOTS-c treatment not only suppressed the production of proinflammatory mediators (iba1, IL-1β, IL-6, TNF-α, iNOS, and BDNF) but also increased the production of anti-inflammatory factors (IL-4, IL-10, and IL-13) and inhibited microglial activation. This finding is consistent with the findings of Wu *et al*. [49] who reported that MOTS-c treatment reduces the mRNA levels of inflammatory cytokines (IL-1β, IL-4, IL-6, and TNF-α) in cardiomyocytes. Similarly, we further investigated whether peripheral macrophages are involved in the therapeutic effects of MOTS-c. MOTS-c treatment markedly inhibited the expressions of CD11b and F4/80, which are markers of macrophages, in tumor-bearing distal femora from BCP mice. Numerous studies have demonstrated that proliferating cancer cells and activated osteoclasts promote the infiltration of macrophages [ 6, 35] . Our data revealed that MOTS-c protected against local bone destruction through the modulation of osteoclast and immune cell function in the tumor microenvironment, providing long-term cancer pain relief.

In addition, MOTS-c was previously shown to improve insulin sensitivity and maintain metabolic homeostasis via the AMPK signaling pathway [ 10, 50] , whereas AMPK has attracted widespread attention as a therapeutic target for regulating chronic pain [ 19– 21] . In particular, a recent report revealed that AMPK activation attenuated cancer-induced bone pain by reducing mitochondrial dysfunction-mediated neuroinflammation [45]. Thus, in this study, we found that treatment with MOTS-c significantly upregulated the phosphorylation of AMPK and that the total AMPK level was not affected. Moreover, compound C, an AMPK pathway inhibitor, was used to evaluate the effects of MOTS-c. Pretreatment with compound C, 30 min prior to injection of MOTS-c, significantly blocked the effects of MOTS-c in mice with BCP. MOTS-c has therapeutic effects on an LLC-induced murine bone cancer model, at least in part through the AMPK signaling pathway. Moreover, according to recent reports, MOTS-c inhibits LPS-induced ERK, JNK and p38 activation in numerous disease models, such as septic cardiomyopathy [49], acute lung injury [51], and cold stress [52]. Unfortunately, in our study, MOTS-c treatment did not alter the BCP-induced increase in phosphorylated p38, ERK, or JNK expression, suggesting that MAPK signaling pathways, including the ERK, JNK, and p38 signaling pathways, are not essential for the effects of BCP.

For the evaluation of MOTS-c side effects, referring to the side effects of common analgesic drugs such as opioid analgesics (morphine) and NSAIDs (celecoxib), our previous studies investigated its antinociceptive tolerance, gastrointestinal transit inhibition, and locomotor function [23]. In this study, we investigated the effects of long-term intraperitoneal injection of MOTS-c on renal function, lipid function, myocardial enzyme profiles, liver function and histological damages to the liver, spleen and kidney. No differences among the groups were observed. In addition, antinociceptive tolerance complicates clinical management strategies and facilitates opioid side effects. Thus, the analgesic tolerance effects of MOTS-c in a bone cancer pain model were evaluated. Our results indicate that there was no development of tolerance to the antinociceptive effects of MOTS-c during 6 consecutive BCP treatments, whereas the antinociceptive effects of morphine were significantly decreased after the fifth treatment. In contrast to morphine-induced analgesia tolerance, MOTS-c produced nontolerance-forming antinociception in BCP.

In summary, the present findings revealed the important role of MOTS-c in the regulation of cancer-induced mechanical allodynia, which is intimately linked to oxidative stress and neuroinflammation via the modulation of the AMPK signaling pathway. Importantly, we discovered that MOTS-c improves BCP via unique peripheral and central synergistic effects on nociceptors, immune cells, and osteoclasts, which provides a pharmacological and biological foundation for developing mitochondrial peptide-based therapeutic agents for cancer-induced pain.

## Supplementary Data

Supplementary data is available at *Acta Biochimica et Biophysica Sinica* online.
